# Local Droplet Etching with Indium Droplets on InP(100) by Metal–Organic Vapor Phase Epitaxy

**DOI:** 10.1021/acs.cgd.4c01097

**Published:** 2024-11-06

**Authors:** Elisa Maddalena Sala, Young In Na, Jon Heffernan

**Affiliations:** 1 EPSRC National Epitaxy Facility, The University of Sheffield, North Campus, Broad Lane, Sheffield S3 7HQ, United Kingdom; 2 Department of Electronic and electrical engineering, The University of Sheffield North Campus, Broad Lane, Sheffield S3 7HQ, United Kingdom

## Abstract

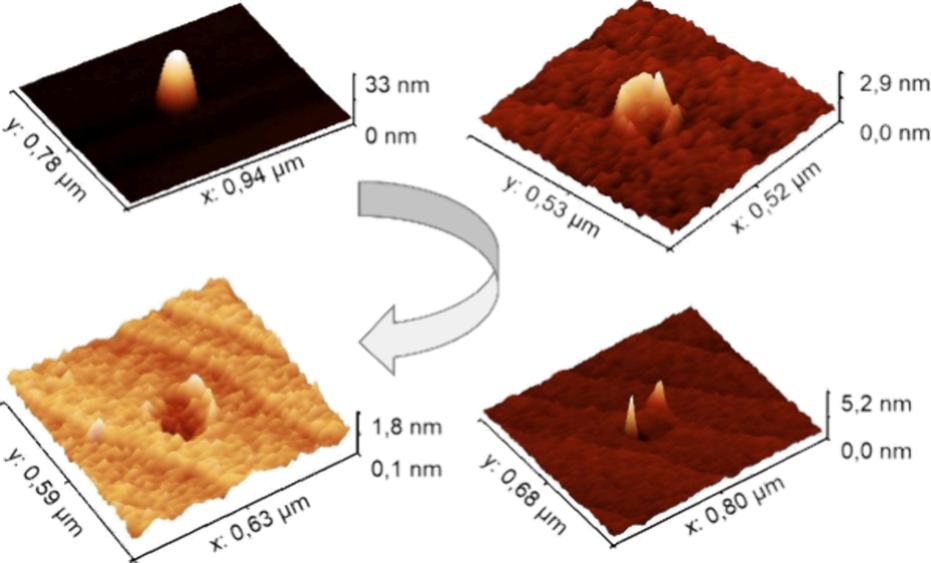

The local droplet etching (LDE) by using indium droplets on bare InP(100) surfaces is demonstrated in a metal–organic vapor phase epitaxy (MOVPE) environment for the first time. The role of an arsenic flow applied to self-assembled metallic indium droplets is systematically studied. Increasing the arsenic supply leads to the formation of ring-like nanostructures and nanoholes. The results are analyzed with reference to LDE in a molecular beam epitaxy environment, where such a technique is well established, particularly for arsenide-based III–V semiconductors, and where only one group-V material is involved. Here, As–P exchange reactions at droplet sites are identified as the drivers for the formation of nanoholes. Such nanoholes can serve as nucleation sites for subsequent fabrication of highly symmetric QDs by nanohole-infilling or as a means for in situ surface nanopatterning. LDE on InP by MOVPE can thus be considered as a promising approach for the cost-effective fabrication of novel quantum emitters at the telecom C-band.

## Abstract 2

As−P exchange reactions at droplet sites are identified as the drivers for the formation of nanoholes. Such nanoholes can serve as nucleation sites for subsequent fabrication of highly symmetric QDs by nanohole-infilling or as a means for in situ surface nanopatterning. LDE on InP by MOVPE can thus be considered as a promising approach for the cost-effective fabrication of novel quantum emitters at the telecom C-band.

## Introduction

Local droplet etching (LDE) is an epitaxial technique used in molecular beam epitaxy (MBE) for the fabrication of self-assembled nanoholes on III–V materials.^1−9^ In-filling and capping of such nanoholes results in 3D quantum confinement in form of highly symmetric and pure quantum dots (QDs) such as GaAs-infilled nanoholes on AlGaAs surfaces.^4,5^ GaAs/AlGaAs QDs fabricated in this way present very interesting physical properties such as a very low fine-structure splitting (FSS) of ∼4 μeV, short lifetime of ∼250 ps compared to ∼1 ns for typical Stranski–Krastanov (SK) InGaAs QDs, and a maximum entanglement fidelity of above 90%.^4^ Such properties render this type of QDs very appealing for applications in quantum information technologies.^5,8^ LDE offers control on the density, size, shape, and symmetry of the resulting QDs via manipulation of the initial droplets and the host material, and it can be also used as an in situ defect-free nanopatterning technique for subsequent localized QD growth,^10,11^ besides other ex situ patterning techniques.^12,13^ It is an associated technique with droplet epitaxy (DE), which is employed for the fabrication of symmetric QDs from metallic group-III droplets and consequent group-V crystallization and allows for a higher degree of freedom in terms of material choice^4^ since it does not rely on strain as is the case for the SK growth mode.^14^ LDE has been extensively studied in MBE mostly for the material system GaAs/AlGaAs targeting the wavelength range of 700–800 nm, compatible with free-space quantum optics applications.^4^ Recent studies by MBE have shown the possibility of droplet etching on other surfaces such as AlGaSb via Ga droplets,^7^ AlInAs via AlIn droplets,^6^ and InGaAs via In droplets^15^ opening the possibility of extending the available wavelengths of the emitters to the telecom range. In this respect, being able to integrate such emitters with the current fiber-based infrastructure is of utmost importance for applications in quantum information technologies, especially for the telecom C-band around 1550 nm.^16^ Moreover, utilizing an epitaxial technique targeting mass production, such as MOVPE, would be beneficial due to reduced fabrication times and costs. After a demonstration of the first quantum light emitting diode (QLED) at the C-band utilizing InAs/InP DE QDs by MOVPE and subsequent qubit teleportation,^17−19^ we recently explored in detail the DE growth of such QDs in the MOVPE environment, studying the nucleation of In droplets and crystallization into QDs both on InP^20−23^ and InGaAs(P) surfaces,^24,25^ and their morphological and optical properties. Here, we study the LDE process in a MOVPE environment on bare InP(100) surfaces. To the best of our knowledge, this is the first systematic study of LDE by MOVPE. We use indium as droplet material and study the effect of the arsenic flow applied to the droplets. We show that As plays a key role in determining the conservation of the total droplet volume, formation of nanoring-like structures, and local droplet etching. As a next step, such nanoholes may be filled in or alternatively treated as nucleation sites for droplets or QDs. The grown material shows very good crystal quality, confirmed by morphological investigations by means of transmission electron microscopy (TEM). Our work opens up further studies on the fabrication of novel quantum emitters by LDE in the telecom C-band, by using a cost-effective epitaxial method transferable to industry.

## Experimental Section

The samples studied in this work were grown in a 3 × 2 close-coupled showerhead (CCS) Aixtron MOVPE reactor using H_2_ as a carrier gas, on on-axis InP(100) substrates. The growth starts with a ∼300 nm InP buffer grown at a substrate temperature of 610 °C. Thereafter, the temperature is decreased to 400 °C for the deposition of indium droplets. Trymethylindium (TMIn) is used as the indium precursor for droplets with a flow of 20 sccm (corresponding to 1.4 μmol/min) for 35 s. Immediately after droplet deposition, arsine (AsH_3_) is supplied for 15 s with a variable flow of 0.1–10 sccm (4.5–450 μmol/min) for 15 s, followed by a 30 s growth interruption (GRI) without any precursor supply. Thereafter, the temperature is ramped to 610 °C while growing the ∼100 nm InP burying layer with a growth rate of ∼0.5 μm/h. For morphological investigations of the free-standing structures, their growth is repeated on top of the InP layer, including the GRI stage, leaving them uncapped. The growth process is then terminated, and the sample is immediately cooled down. The samples are characterized morphologically with atomic force microscopy (AFM) for the surface structures, with TEM for the buried ones, and optically via room- and low-temperature photoluminescence (RT-PL, LT-PL). For RT-PL, a 645 nm diode laser with 85 W·cm^–2^ power density was used, while for LT-PL, the samples were cooled down at 4 K and measured with a 635 nm laser with a power density of 450 W·cm^–2^. TEM micrographs were taken under (002) dark field conditions, which allowed the layer structures and compositional variations to be distinguished via dark/bright contrast. Such conditions are also partly sensitive to strain fields in the specimen.^26^

## Results and Discussion

**Figure 1:**
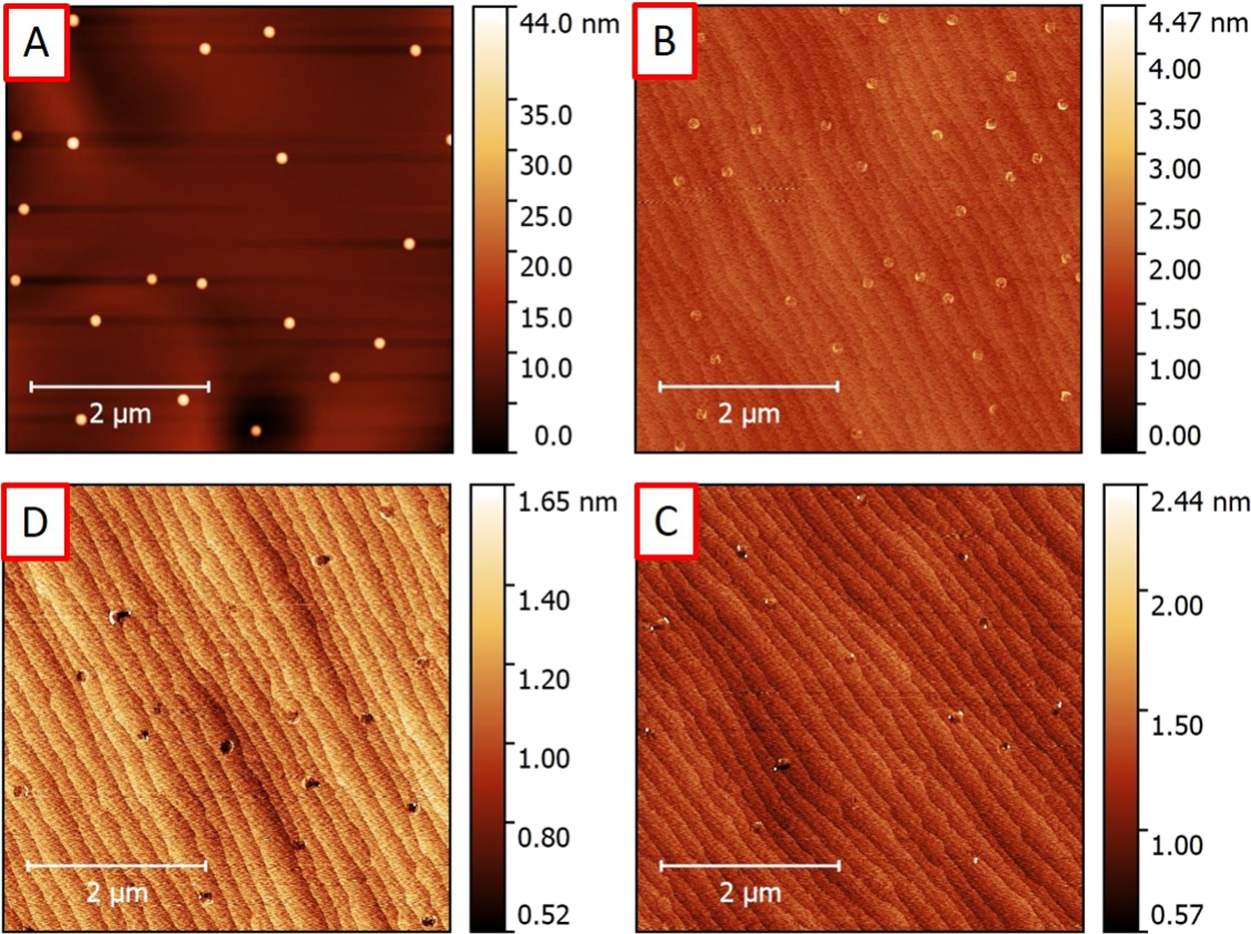
5 × 5 μm^2^ AFM micrographs of the four samples studied in this work corresponding to increasing As flow from 0.1 to 10 sccm (A–D).

**Figure 2:**
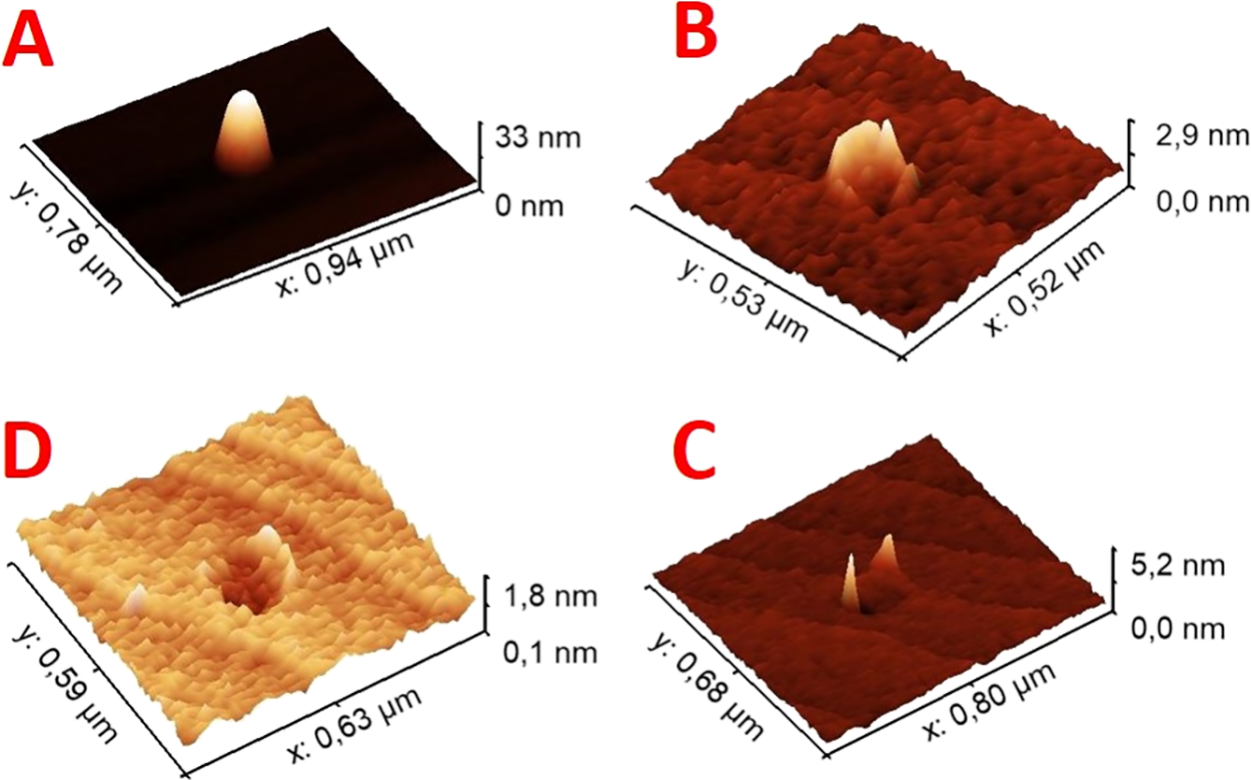
3D AFM micrographs of selected structures from samples A–D with variation of As supply: (A) 0.1 sccm: indium droplet, (B) 0.5 sccm: nanoring-shaped structures, (C) 3 sccm: initial formation of etch pits with thin walls, and (D) 10 sccm: etch pits with shallow walls.

We studied the effect of a variable AsH_3_ flow applied to an ensemble of indium droplets at a temperature of 400 °C deposited directly on bare InP(100) surfaces. Figure 1 shows AFM micrographs of samples with droplets exposed to AsH_3_ flows of (A) 0.1, (B) 0.5, (C) 3, and (D) 10 sccm, respectively. We identified four regimes depending on the AsH_3_ flow used, i.e., (A) conservation of total droplet volume, (B) crystallization into nanoring-shaped structures, (C) etch pits with partially crystallized walls, and (D) etch pits with very shallow walls. Three-dimensional AFM micrographs of selected structures corresponding to those shown in Figure 1 are presented in Figure 2 for better visualization of their morphology.

**Figure 3:**
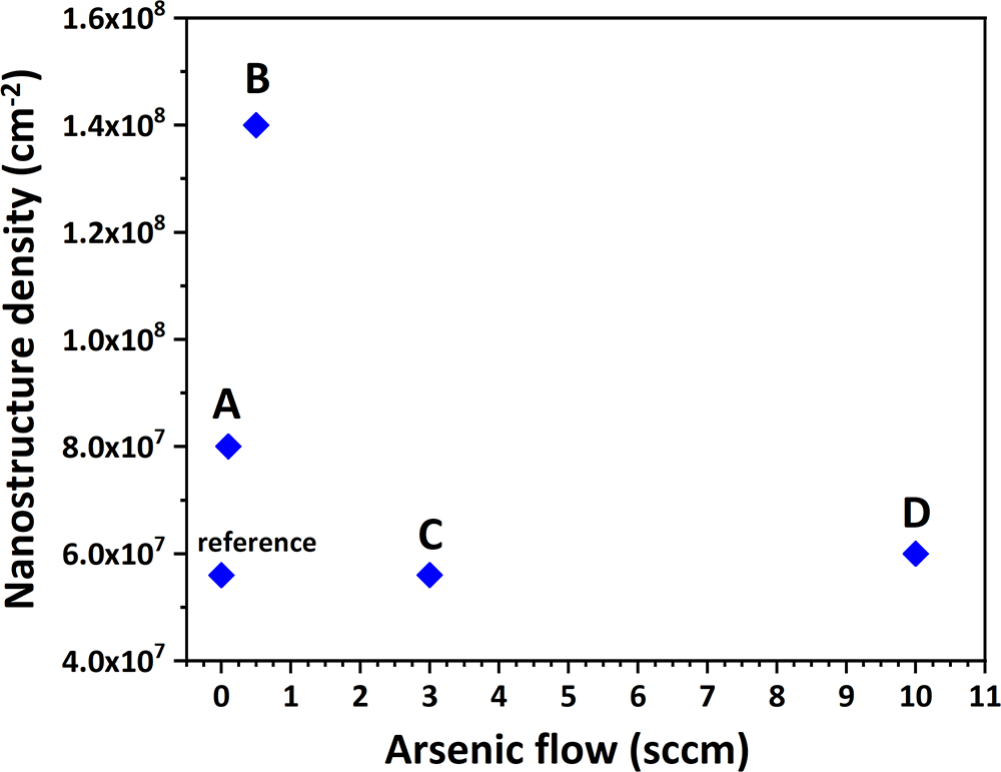
Densities of the nanostructures with varying arsenic flow for samples A–D and the reference sample without an arsenic supply.

We first observe that the density of the droplets exposed to a very low AsH_3_ flow in Figure 1A has slightly increased from ∼5.6 × 10^7^ to ∼7.8 × 10^7^ cm^–2^ compared to droplets deposited with the same growth conditions but without any AsH_3_.^20^ Also, the droplets now appear smaller, with a width of (172.6 ± 6.6) and height of (32.4 ± 1.2) nm, decreasing from the original width of (187.0 ± 7.2) and height of (40.0 ± 1.5) nm for the droplets without AsH_3_. The conservation of the total droplet volume was checked when droplets were exposed to AsH_3_. The volume corresponds to (3.7 ± 0.9) × 10^13^ and (3.5 ± 0.5) × 10^13^ nm^3^/cm^2^ for droplets with and without AsH_3_, respectively. Thus, we can confirm that the total volume of the deposited indium is conserved, lying within the error margin. The variations observed in droplet size and density in the presence of AsH_3_ suggest that the arsine supply affected the indium surface diffusion, resulting in a slightly increased droplet nucleation (higher density and reduced size). This points to a reduction of the indium diffusion in the presence of As, a well-known phenomenon described as the poisoning of surface nucleation sites by AsH_3_, which indeed hinders In diffusion.^27^ Unlike in MBE, where the group-III element comes from molecular flows and has a higher sticking to the surface (corresponding to one at usual growth conditions^28,29^), in a MOVPE environment the availability of the atomic indium depends on the decomposition of its precursor TMIn occurring at the surface.^30,31^ This means that only when the indium atoms are released from the methyl ion groups can they nucleate on the surface and coalesce into droplets. Thus, upon TMIn deposition, droplets are not formed instantly. Here, immediately after TMIn deposition, AsH_3_ is supplied, and this will affect the surface chemistry and thus the droplet nucleation and their density. The density of the structures in Figure 1B increases from the droplets in Figure 1A, i.e., from ∼7.8 × 10^7^ cm^–2^ to ∼1.4 × 10^8^ cm^–2^, whereas for the structures in Figure 1C, the density drops to ∼6 × 10^7^ cm^–2^, remaining approximately the same for the etched pits in Figure 1D, i.e., ∼6 × 10^7^ cm^–2^. In Figure 3, we summarize the variation in density of the nanostructures for samples A–D, depending on the arsenic flow applied. The observed sharp increase in density for sample B (0.5 sccm AsH_3_) can be explained by further surface poisoning by the additional AsH_3_, the phenomenon also discussed above.^27^ Upon additional arsenic deposition from 0.5 to 3 sccm (sample C) an abrupt decrease in density is observed. The presence of more hydride species here enhances the TMIn decomposition since they act as getters of the radicals produced in the TMIn decomposition reactions.^30^ This will likely increase the availability of indium on the surface and affect (i.e., increase) its surface diffusion,^27^ thus reducing the density of the nanostructures. Here, it is worth noticing that the droplet deposition temperature is 400 °C, which is below the full pyrolysis temperature for TMIn (approximately 480 °C^32^). Therefore, the hydride species have a chance to enhance the TMIn decomposition.

Further increasing the As flow for D (from 3 to 10 sccm) does not appreciably affect the density of the structures, possibly due to the fact that the indium has already fully pyrolyzed with the help of hydride species.

**Figure 4:**
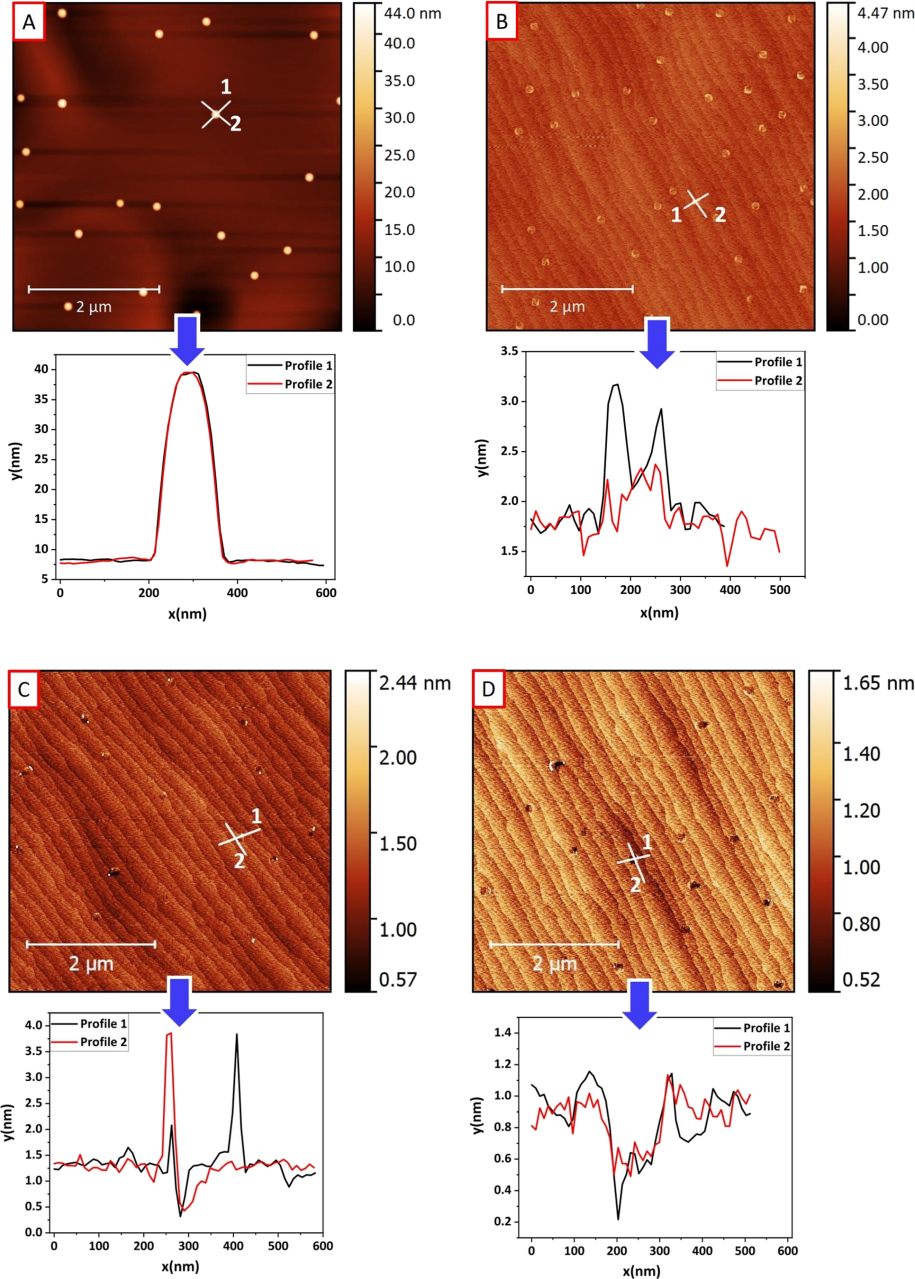
AFM micrographs and profiles of selected structures of samples A–D.

Figure 4 displays profiles of selected etched pits, as shown in Figure 1. We observe conservation of total droplet volume in A, whereas in B, a clear crystallization of walls at the side of the droplets is present, forming a ring-like structure, but without any etching occurring. In C, the walls appear reduced in height and the etching becomes observable, with a depth of ∼0.5 nm. In D, the walls become shallower, although not completely disappearing, with an etching ∼0.6 nm deep. Clearly, we observe here the formation of etched nanoholes in both samples C and D.

**Figure 5:**
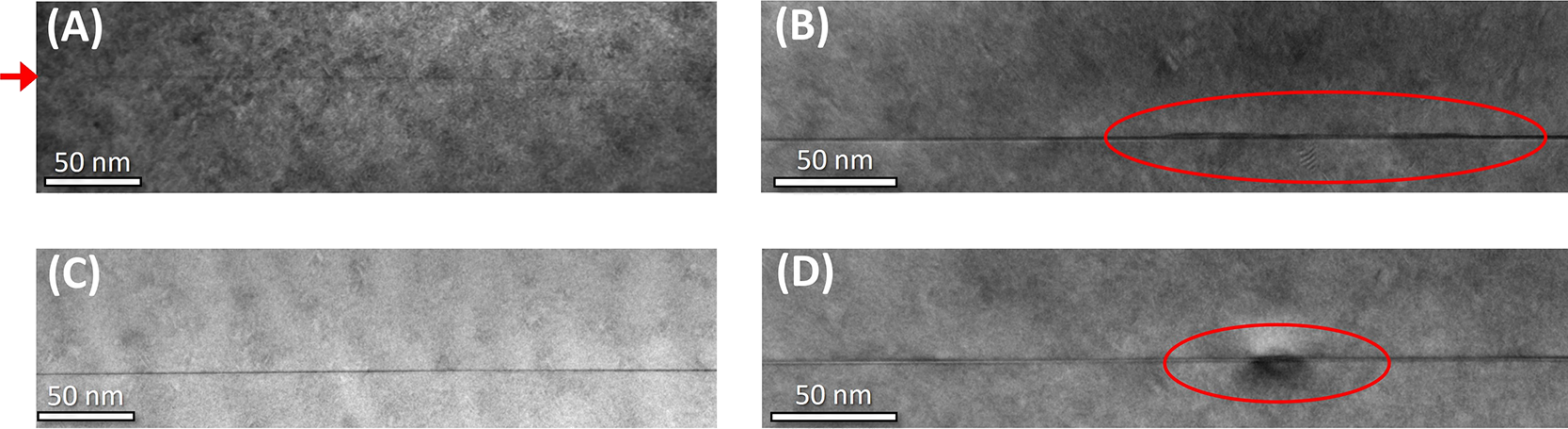
TEM micrographs of samples A–D.

Here, it is clear that the variation of the As flow drives the evolution of the LDE process, from droplet to nanohole etching and formation of nanorings. To investigate these effects further, we carried out TEM and PL measurements. Figure 5 shows TEM micrographs corresponding to the four structures in Figure 1 but where crystallized droplets were buried by a ∼100 nm InP layer. Here we note that TEM has been carried out over large areas of the samples, thus we can confidently confirm that these micrographs are realistic representations of the actual layer structure. In all four samples, a dark line emerges from the background, which we attribute to an InAs_*x*_P_1–*x*_ 2D quasi-wetting layer (WL), which is commonly observed for DE of InAs/InP QDs and created via As/P exchange reactions during the exposure of the InP surface to the AsH_3_ flow.^13,20−23^ Such reactions are activated by temperature and depend on the applied AsH_3_ flow.^33−36^

**Figure 6:**
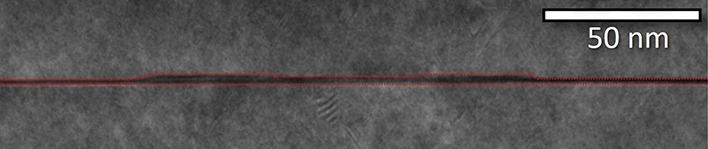
TEM micrograph of sample B. The red dotted lines highlight the profile of the capped ring structure.

We observe slight thickness variations of this 2D layer with the varying As flows used across samples. In particular, for sample A, corresponding to the minimized As flow of 0.1 sccm, the layer appears rather faint, see red arrow for better visualization, whereas for samples B–D, it is more well-defined showing a darker contrast. The thickness of the 2D layer has been estimated from these TEM micrographs by using line profiles. For sample A, the line is very thin, less than 0.5 nm. We note that for this sample, in particular, it is difficult to provide a precise measurement of such a thin layer with TEM, as it is likely not a homogeneous closed layer. As mentioned above, the As exchange is a process depending on temperature and applied AsH_3_ flow^33−37^ and the current temperature of 400 °C is just above the threshold (350 °C) to provide sufficient activation energy for the As/P exchange process to proceed.^37^ Generally, at 400 °C and with such a low arsenic flow (0.1 sccm) in MOVPE conditions, the exchange is expected to be less than a ML—for bare InP surfaces exposed to As.^36^ However, here we note that a certain indium wetting is present on the InP surface (due to the indium droplet deposition), which can promote the formation of the InAs_*x*_P_1–*x*_ 2D layer by acting as a group-III reservoir. It was indeed demonstrated that the indium wetting contributed to the formation of such a layer in our previous experiments.^20−23^ Thus, its thickness could be slightly increased compared to what is expected for InP bare surfaces, as discussed above. In B, we observe thick regions, which are highlighted by red circles, showing a maximum of ∼2.6 nm height and ∼130 nm length. These are also shown in more detail in Figure 6: two ∼2.6 nm areas appear to be bound with a thinner line ∼1.4 nm thick and 40 nm long. The 2D-layer areas outside the ring region instead display an approximate thickness of ca. 0.8 nm. The size of the structure displaying the two thicker areas bounded together corresponds to what was observed via AFM above, and it is attributed to a typical ring-shaped structure as seen in Figures 1B and 2B. In this TEM micrograph, we do not observe an appreciable dark/bright contrast between the 2D layer and the overlaying ring structure, indicating no substantial change in composition. However, we note that the area under consideration is rather thin, and thus, a clear contrast under the current TEM conditions is hard to distinguish. Therefore, a compositional variation between the 2D layer and the ring may go undetected.

In sample C, no ring-like structures can be detected in TEM, also inspecting larger areas of the sample. Here, more Arsenic is used than in sample B (3 vs 0.5 sccm). This likely leads to an increased As content in the crystalline structures compared to B. This would point to an increased local lattice mismatch upon capping with InP. As observed for other material systems such as InAs/GaAs, the higher the lattice-mismatch, the more enhanced is the nanostructure decomposition upon capping. This also causes a material redistribution not following the usual route from apex to base of the nanostructure^38^ but into the wetting layer.^39^ Thus, here, the demolished As-rich material becomes part of the 2D layer. Its thickness for C is estimated to be in the range of 0.4–0.9 nm, where the layer shows slight thickness modulations, whereas for D, it lies within the range of 1–1.3 nm. Additionally, a region with a dark round halo extending locally underneath and above the 2D-layer line is detected, see Figure 5D, which is not present in any of the other samples. Most likely, this is due to a locally increased strain field corresponding to the crater positions. The (002) dark field conditions are indeed partially sensitive to strain fields, thus allowing to reveal local strain in the structures.^26^ Here, the nanohole structures are buried, meaning they are in-filled with InP during the capping process. They are also the largest in diameter, and the highest amount of arsenic is used for this sample, which will result in an arsenic-richer interface. From these observations, we can infer that a local increase in strain field is generated at crater sites during the InP overgrowth. For samples A and C, no 3D structures nor strain fields are detectable in TEM. In A, the liquid indium from the droplets on the surface has likely spread out on the surface and then become part of the InP burying layer upon PH_3_ supply. Finally, we point out that the analyzed samples possess an overall very good crystalline material quality as no defects or dislocations are detected.

**Figure 7:**
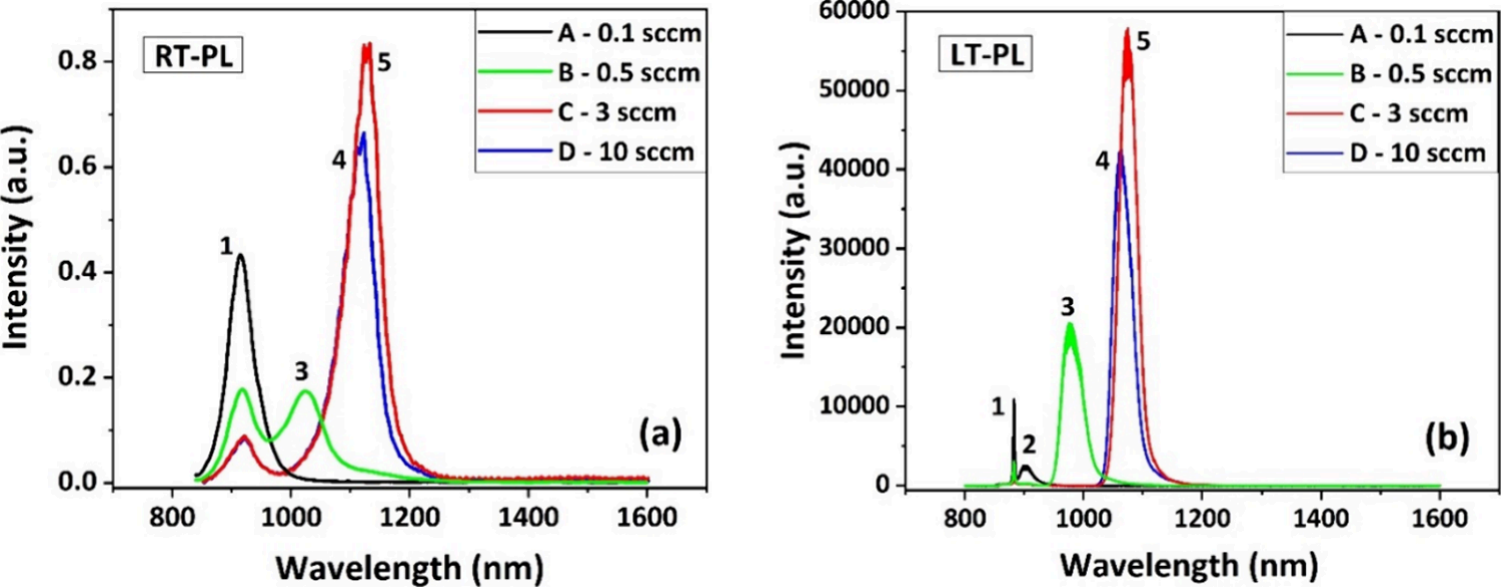
PL spectra of all investigated samples recorded at (a) room temperature and (b) at 4 K.

Figure 7 shows photoluminescence spectra taken at both (a) room temperature and (b) low temperature (4 K) on all four samples. The PL spectra were fitted with Gaussian curves (not shown here). The emission labeled as **1** is visible for all samples and corresponds to InP^40^: this is around 920 nm at RT (a) and 882 nm at LT (b). Sample A shows an additional weak emission at 903 nm LT-PL only, labeled with **2** in Figure 7b, which we ascribe to the emission from the initial very thin InAs_*x*_P_1–*x*_ 2D layer observed in TEM of Figure 6A. Based on detailed PL studies and theoretical modeling of strained InAs_*x*_P_1–*x*_ quantum wells (QWs) with different thicknesses and As/P ratios grown by MOVPE,^41^ we estimate an arsenic fraction between 30 and 40% if we consider a thickness of maximum 0.5 nm (from our TEM estimate) for this layer in sample A.

**Figure 8:**
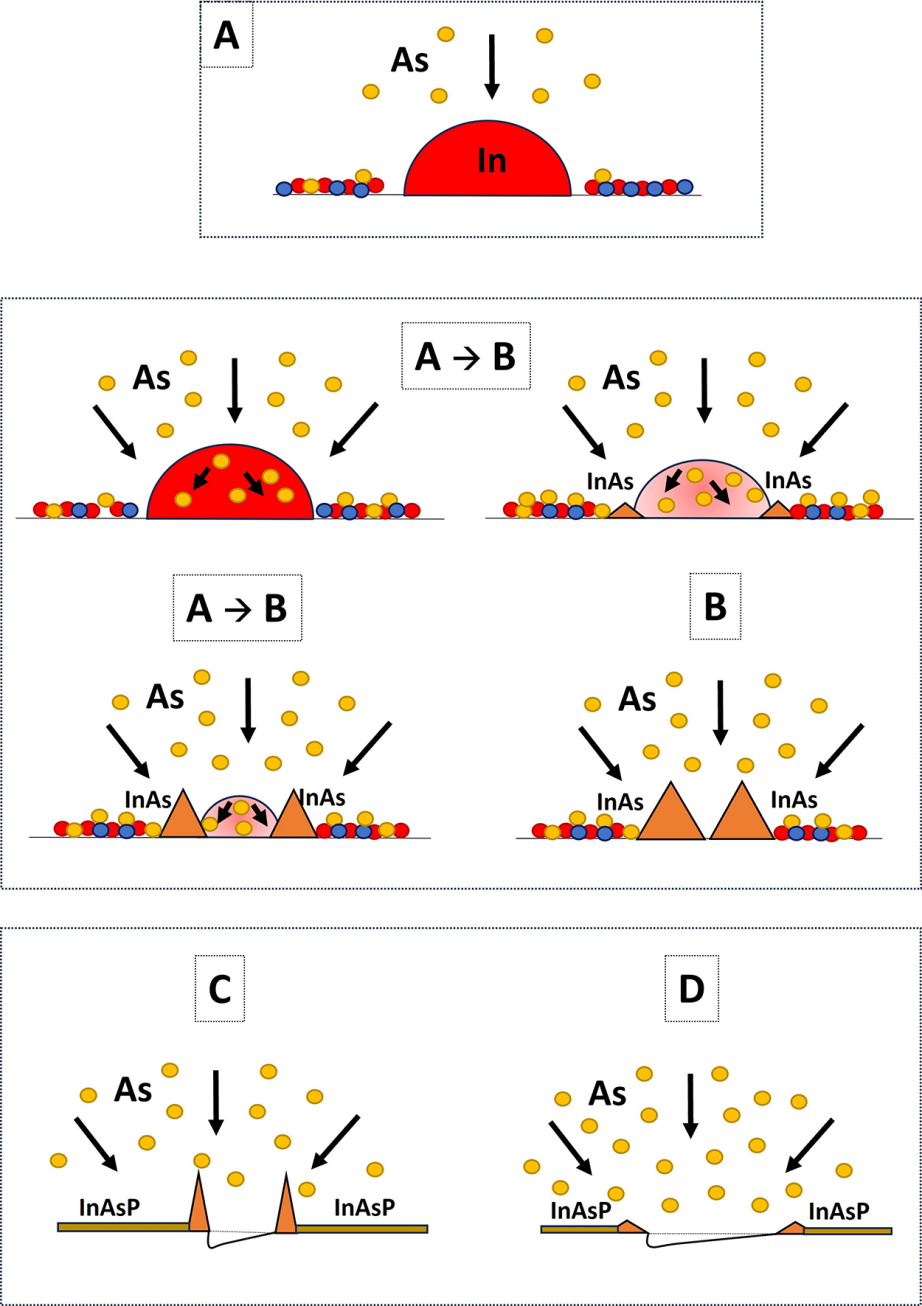
Sketch of the stages of the LDE process from droplet (A) to nanohole formation (C, D) through ring formation (B). The circles represent the atoms involved in the process as follows: As (yellow), In (red), and P (blue). The orange triangles are the InAs crystallized structures.

The fact that such emission quenches at RT confirms that this layer is very thin, thus it does not represent an efficient recombination channel for carriers at RT, as also seen elsewhere.^41^ Sample B shows a more defined emission from the 2D layer, which is red-shifted compared to the one of sample A, labeled with **3**, emitting around 1021 nm at RT (a) and at 981 nm at LT (b). In samples C and D, such emission redshifts further to around 1123 and 1113 at RT and to 1075 and 1066 nm at LT; for C and D, see peaks labeled **4** and **5**, respectively. It also becomes brighter, indicating better carrier capture. Here the arsine flows used are 3 and 10 sccm for samples C and D, respectively. Based on the theoretical predictions for PL emission of InAs_*x*_P_1–*x*_ QWs with different As/P ratios and thicknesses,^41^ we can confirm that the thickness variations (overall less than 1 nm) of the 2D layer seen here alone do not justify the significant redshift of the PL. In fact, we do not expect to observe substantial variations in the 2D-layer thickness as the total amount of deposited indium forming droplets and wetting the InP surface has not changed among the samples. We thus conclude that the increased arsenic incorporation in the layer significantly contributes to the observed redshift. For samples C and D, the arsenic content could exceed 50%, based on the theoretical estimates.^41^ The fact that the spectral positions for C and D remain substantially unchanged indicates a saturation of the arsenic incorporation into the 2D layer, which is expected for high arsenic flows.^33^ Finally, we can exclude that the bright emissions **4** and **5** originate from the nanorings, since for C and D, they have disappeared leaving craters and only very thin walls around them, see AFMs of Figure 4. Additionally, the brightness of both **4** and **5** has increased considerably compared to **2** and **3**, pointing to a stronger recombination channel for the photogenerated carriers, which is most likely provided by a more uniform 2D layer. Considering the previous discussion on AFM, TEM, and PL of the four samples studied in this work, in the following we describe the four stages of the ring formation and LDE processes (A to D). We analyze the results with reference to such processes in the MBE environment. We also refer to the sketches in Figure 8 for a better visualization of each step.

In MBE, the etching occurs when the droplets are annealed under low group-V overpressure and the substrate surface underneath the droplets liquefies.^3,4,7,9^ In the current MOVPE conditions, since the liquid In and the InP substrate are at stable phases (temperature is well below 500 °C),^22,42,43^ InP dissolution in In and P atoms is not expected. Also, contrary to MBE, where LDE is carried out in the presence of only one group-V—either arsenic^2−6,15^ or antimony^7^—the presence of two group-V elements here, namely, As and P, adds an additional degree of complexity to the understanding of the LDE mechanism. We thus conclude that a modified etching dynamic involving another process drives the actual etching, in our case, in an MOVPE environment.

a. Indium droplets, with total volume conservation under minimized As flow, see Figure 8A. Here, a balance between indium atom detachment from the droplet and reattachment to the droplets is struck and the total droplet volume is conserved without droplet crystallization. This stage resembles what is usually observed under MBE conditions during the early stages of LDE, under minimized As background pressure.^3,4,7,9^ There, the volume of the single droplets is conserved, differently from the present case, where the total volume is conserved, but the volume of each droplet varies. As discussed above, in this case, the presence of AsH_3_ does affect the In surface diffusion during In droplet nucleation, so that a slightly higher droplet density with reduced size is observed. Overall, this has to be considered as a dynamic process with indium atoms diffusing, coalescing, attaching, and detaching from droplets while maintaining the droplets themselves intact and with no etching occurring. At the same time, an initial very thin 2D layer is formed via As/P exchange, which is confirmed by the TEM and LT-PL studies discussed above. The profile of a typical droplet is shown in Figure 4A, showing a symmetric shape.
b. Formation of (inner) InAs nanorings without etching underneath the droplet—intermediate state. The arsenic from the ambient now starts diffusing into the droplets increasing its concentration and causing supersaturation. Due to the low solubility of the As in the indium droplet, and the convection flux internal to the droplet, a localized InAs growth at the internal edges of the original droplet takes place, similarly to what was observed in MBE.^45,46^ Looking at the example profile in Figure 4B, the structures present a width of (164.0 ± 22.8) nm and a height of (1.47 ± 0.75) nm, where a reduction in both width, and especially height, from the original droplet is observed, which were (172.6 ± 6.6) and (32.4 ± 1.2) nm, respectively. We note that the average reduction in lateral size from the droplets in A to the InAs ring structures in B is just ∼5%, thus confirming the formation of the ring structures within the droplet perimeter: these are also referred to as inner rings.^44−47^ The formation of ring-like structures without etching is a result of the interplay between two main factors, that is the applied arsenic flow and substrate temperature.^44−47^ It is well-known that for the DE technique in MBE, a variety of nanostructures can be fabricated by varying these two growth conditions. Such structures include QDs, rings, as well as double-ring structures. Generally, complex kinetics models are used to describe ring formation in an MBE environment,^44,48^ and a detailed modeling of this mechanism in MOVPE currently goes beyond the scope of this work. A theoretical model based on real-time observations of ring formations under MBE conditions describing the stages of various stages and types of rings can be found in ref (44).
c. Actual droplet etching. Here, the increase in arsenic flow to 5 sccm marks the start of the transition to the actual etching process. We clearly observe etched nanoholes on the InP surface with thin InAs crystalline wall structures on their side, see also Figure 4C and sketch in Figure 8C. The width of the nanoholes is (169.8 ± 35.1) nm with a depth of (0.53 ± 0.17) nm. We note that this is an actual etch, as the typical surface roughness for a layer-by-layer grown InP is just ∼150 ppm.^49^ The height of the crystalline walls is (3.5 ± 1.2) nm, increased compared to sample B, whereas only minor variations in the lateral size of the craters are observed. As noted above, the etching process here has a different driving force compared to LDE in MBE where only one group-V is involved. Most likely, when the As concentration in the droplets further increases due to the higher As flow supplied, As/P exchange reactions have the chance to occur underneath and around the droplets, helping to release P atoms, thus dissolving the InP surface locally and creating a nanohole. In LDE by MBE for GaAs, the increase in As flow leads to an imbalance in the equilibrium composition of the droplet which drives more Ga atoms from the substrate into the droplet to compensate for it.^2,3,7,9^ This enhances the substrate dissolution locally resulting in etching. We previously observed substrate etching around InAs/InP DE QDs, which was driven by InP surface destabilization strictly at temperatures >500 °C, followed by indium migration toward the droplet.^20,22^ No etching was observed during arsenic supply for temperature below 500 °C even at prolonged arsenic supply nor increased As flows. We can thus conclude that the driver for the etching here is given by As/P exchange reactions. The P atoms released by the As/P exchange can travel through the droplet and are released back into the growth chamber.^22^ Since the As/P exchange is self-limited to the upper few MLs, and strongly dependent on temperature,^33−37^ the depth of the etching here is limited to less than 1 nm.^35^ This contrasts with what occurs in MBE for GaAs- and GaSb-based systems, where the typical depth of nanoholes is a few tenths of nanometers.^2−7,9,48^ We note that the round inner nanorings have now disappeared leaving only sharp thin wall structures, although still being positioned within the original droplet diameter, as for B. The indium atoms detaching from the original droplets crystallize in the presence of the As flow partly forming the sharp, but thin, InAs walls and additionally contribute to the formation of the 2D layer. As discussed in the PL investigations above, the emission from the 2D layer has red-shifted for this sample, see Figure 7, and become brighter, indicating a higher As/P compositional ratio and a better carrier capture into the layer (we note that, as detected by TEM, its overall thickness has not appreciably changed from B). From these observations, we can conclude that the presence of more arsenic drives the LDE. We finally note that the etching is asymmetric (with the nanohole placed at one corner of the original droplet position). This is expected and also observed in LDE in MBE, where the progressive droplet consumption etches down the substrate at one preferential side.^50^
d. Etching with shallow (outer) shallow InAs nanorings. Here, the lateral size of the etched holes has increased to a width of (201.7 ± 28.9) nm, while the height of the remaining InAs rings decreased to (1.38 ± 1.06) nm from (3.5 ± 1.2) nm of the structures in B. The craters are now on average larger than the original droplets deposited without arsenic, which were (187.0 ± 7.2) nm wide. The depth of the etching has only slightly increased to (0.59 ± 0.19) nm and remains in the same order of magnitude compared to sample C, see also Figure 4D. The density is comparable to the structures in C. In B, we discussed the formation of inner InAs nanorings with diameters lying within the original droplet area. During ring formation in MBE conditions, either structures with single (inner), double (outer), or concentric rings are formed, depending on the growth conditions (As supply and substrate temperature) and oftentimes, when a single ring is observed, this is due to an overlap of double rings.^45−47^ The interplay of the As flow and substrate temperature results in the formation of either one of the above structures.^45−47^ The outer ring is usually formed at the periphery of the original droplet, displaying lower crystalline walls compared to the structures having only the inner rings, and it is the result of reactions between the group-III atoms out-diffusing from the droplet area on the surface with the group-V atoms diffusing inward (toward the droplet) over a reaction-diffusion zone.^45^ Here, we attribute the low rings in D to the outer rings formed around the droplets upon increased arsenic flow. As seen in C, an etch pit is observed also in this case, being slightly deeper on average, and this is again attributed to the As/P exchange reactions driving the etching process.

## Conclusions

In conclusion, we demonstrated the formation of nanorings and local droplet etching by indium on bare InP (100) surfaces in an MOVPE environment for the first time. We explored the effect that arsenic has on the processes, showing that it plays an important role in determining the type of nanostructure and the magnitude of the etching underneath the initial droplets. Nanorings are formed with an arsenic flow of 0.5 and 3 sccm, where 3 sccm kickstarts the actual etching, and finally, increasing the flow up to 10 sccm leaves bigger craters with outer shallow nanorings formed at the original droplet periphery. Such nanoholes could be employed as nucleation sites for subsequent localization of droplets and QDs, or for further fabrication of highly symmetric QDs by in-filling. We thus showed that such a technique can be employed to form various kinds of nanostructures, specifically nanorings, and as an in situ defect-free nanopatterning technique in a MOVPE environment, similar to MBE. Morphological characterizations by TEM confirmed a high crystalline quality of the epitaxial material, which appears homogeneous without defects or dislocations. Optical investigations suggest a dynamic evolution of the InAs_*x*_P_1–*x*_ 2D-layer growth with the applied arsenic. As the next steps, additional MOVPE growth conditions will be explored in order to study their effect on the nanohole formation, size, and shape, to target the right conditions for in-filling to obtain highly symmetric QDs. The LDE mechanism can also be coupled to site-control approaches to obtain defect-free nanohole arrays for the subsequent site control of QDs. This study thus opens up the use of LDE in MOVPE for the fabrication of novel InP-based telecom C-band emitters for quantum communication technologies.
